# Buwchitin: A Ruminal Peptide with Antimicrobial Potential against *Enterococcus faecalis*

**DOI:** 10.3389/fchem.2017.00051

**Published:** 2017-07-12

**Authors:** Linda B. Oyama, Jean-Adrien Crochet, Joan E. Edwards, Susan E. Girdwood, Alan R. Cookson, Narcis Fernandez-Fuentes, Kai Hilpert, Peter N. Golyshin, Olga V. Golyshina, Florence Privé, Matthias Hess, Hilario C. Mantovani, Christopher J. Creevey, Sharon A. Huws

**Affiliations:** ^1^Institute of Biological Environmental and Rural Sciences, Aberystwyth University Aberystwyth, United Kingdom; ^2^Institute of Infection and Immunity, St George's University of London London, United Kingdom; ^3^School of Biological Sciences, Bangor University Bangor, United Kingdom; ^4^College of Agricultural and Environmental Sciences, University of California, Davis Davis, CA, United States; ^5^Department of Microbiology, Universidade Federal de Viçosa Viçosa, Brazil; ^6^Medical Biology Centre, School of Biological Sciences, Queen's University Belfast Belfast, United Kingdom

**Keywords:** microbiome, metagenomics, rumen bacteria, antibiotic resistance, antimicrobial peptides, antimicrobial activity, *Enterococcus faecalis*

## Abstract

Antimicrobial peptides (AMPs) are gaining popularity as alternatives for treatment of bacterial infections and recent advances in *omics* technologies provide new platforms for AMP discovery. We sought to determine the antibacterial activity of a novel antimicrobial peptide, buwchitin, against *Enterococcus faecalis*. Buwchitin was identified from a rumen bacterial metagenome library, cloned, expressed and purified. The antimicrobial activity of the recombinant peptide was assessed using a broth microdilution susceptibility assay to determine the peptide's killing kinetics against selected bacterial strains. The killing mechanism of buwchitin was investigated further by monitoring its ability to cause membrane depolarization (diSC_3_(5) method) and morphological changes in *E. faecalis* cells. Transmission electron micrographs of buwchitin treated *E. faecalis* cells showed intact outer membranes with blebbing, but no major damaging effects and cell morphology changes. Buwchitin had negligible cytotoxicity against defibrinated sheep erythrocytes. Although no significant membrane leakage and depolarization was observed, buwchitin at minimum inhibitory concentration (MIC) was bacteriostatic against *E. faecalis* cells and inhibited growth *in vitro* by 70% when compared to untreated cells. These findings suggest that buwchitin, a rumen derived peptide, has potential for antimicrobial activity against *E. faecalis*.

## Introduction

*Enterococcus faecalis* is a non-motile, Gram-positive, facultative anaerobic lactic acid bacterium of about 0.6–2.0 μm in size, that grows as individual cells, in pairs or as short multicellular filaments (Leavis et al., 2006; Ch. Schroder et al., 2015). It tolerates a wide variety of growth conditions, including temperatures between 10 and 45°C, hypotonic, hypertonic, acidic, or alkaline environments (Ch. Schroder et al., 2015). *E. faecalis* is normally a gut commensal found in many animals and in the environment (Gilmore et al., 2013). It is also a frequent cause of many serious human infections, including urinary tract infections, endocarditis, bacteremia, and wound infections alongside *Enterococcus faecium* (Kau et al., 2005; Gilmore et al., 2013; Cahill and Prendergast, 2016). *E. faecalis* causes a variety of healthcare associated infections of which urinary tract infections are the most common (Kau et al., 2005; Hidron et al., 2008; Arias and Murray, 2012; Gilmore et al., 2013). Infections with *E. faecalis* can be especially challenging to treat because of their frequent resistance to multiple antibiotics, including aminoglycosides, and vancomycin, which is considered as drug of last resort for many Gram-positive infections (Baddour et al., 2005; Hollenbeck and Rice, 2012; Young et al., 2016). Vancomycin-resistant enterococci (VRE) are significant opportunistic pathogens in the hospital environment and often possess a multidrug-resistant phenotype (Chavers et al., 2003; van Harten et al., 2017) and their potential to spread enterococcal vancomycin resistance to other species remains a concern (Chang et al., 2003). VRE are also listed as priority pathogens by the World Health Organization for research and development of new antibiotics (WHO, 2017). It is therefore important to develop new drugs for the treatment of enterococcal infections.

Continued development of new drugs by the pharmaceutical industry, aided by genomics, high-throughput screening, rational drug design, and novel therapies offer a very promising prospect of effective bactericidal monotherapy for Enterococci and long-term solutions to VRE (Eliopoulos and Gold, 2001). Antimicrobial peptides (AMPs) are an integral part of the innate host defense system of many organisms including vertebrates, invertebrates, plants and bacteria (Wiesner and Vilcinskas, 2010), with broad spectrum activity against several groups of organisms including multidrug resistant bacteria, fungi, viruses and parasites (Jenssen et al., 2006). Due to this, AMPs represent one of the most promising alternatives to antibiotics, and future strategies for defeating the threat of antimicrobial resistance in bacterial infections might depend on peptide-based antimicrobial molecules (Czaplewski et al., 2016; O'Neill, 2016).

The rumen is one of the most diverse ecosystems in nature, harboring a microbial community, composed of a complex mixture of bacteria, protozoa, fungi, and viruses (Church, 1993; Sirohi et al., 2012) commonly referred to as the rumen microbiome, and enzymes isolated from this ecosystem have the potential to possess very unique biochemical properties (Hess et al., 2011; Ross et al., 2012). Several ruminal bacteriocins have been identified to date, but all of these bacteriocins are derived from bacteria that can be grown in the laboratory (Russell and Mantovani, 2002; Azevedo et al., 2015). Culture independent methods can be used to assess the rumen microbiome and increase the repertoire of bacteriocins, and other novel antimicrobials. It is possible to access and explore the total genetic information of this underexplored, uncultured fraction of the microbiome associated with any defined ecosystem through the application of metagenomics (Handelsman et al., 1998; Ekkers et al., 2012), which is the analysis of the DNA from a microbiome. Direct cloning of genomic or metagenomic DNA also offers the opportunity to capture genes encoding the synthesis of novel antimicrobials (Schloss and Handelsman, 2003), whether from species with already known antimicrobial properties (bacteriocin production), or from completely new species.

Previously, we prospected a 8,448 clone fosmid-based rumen bacterial metagenomic library generated from cow rumen solid attached bacteria (SAB) for novel antimicrobials, combining both functional and sequence based metagenomics and *in silico* mining (Oyama, 2015; Prive et al., 2015). From this work, we identified numerous AMPs and mini proteins. Results of the activity screens of the identified short AMPs (≤25 AA) were reported elsewhere (Oyama, 2015). One of the longer proteins, buwchitin (71 AA) was selected for further characterization due to its potential activity against *E. faecalis*. In this study, we report the potential antimicrobial activity of buwchitin against *E. faecalis*.

## Materials and methods

### Bacterial strains and vectors

Bacterial strains used for antimicrobial activity testing were provided in-kind by Bath University. Strains include methicillin sensitive *Staphylococcus aureus* (MSSA) RN4220, *Escherichia coli* K12, *Salmonella enterica* serovar Typhimurium SL1344, *Listeria monocytogenes* NCTC 11994 (serovar 4b) and *Enterococcus faecalis* JH2-2. *E. coli* TOP10 (Invitrogen, Carlsbad CA, USA) was used for cloning (to host expression vectors for protein expression). The pTrcHis TOPO® vector (Invitrogen, Carlsbad, CA, USA) was used to clone polymerase chain reaction (PCR) products for protein expression.

### Bacteriological media and culture conditions

Mueller Hinton (MH) (Sigma-Aldrich UK) and Luria Bertani (LB) broth and agar (Fisher Scientific Leicestershire, UK) were used as growth media. When leakage assays were performed under buffered conditions, 5 mM HEPES (pH 7.2) supplemented with 5 mM glucose was used (Wu and Hancock, 1999). Media were prepared and sterilized according to the manufacturers' instructions. Bacterial strains were grown using standard conditions unless otherwise specified. Broth cultures were incubated at 37°C for 18–20 h with aeration and cultures on solid media were incubated at 37°C for 18–24 h.

### Identification of antimicrobial genes from fosmid metagenomic library by agar based functional screening and sequencing analysis

Antimicrobial genes were identified from the fosmid metagenomic library as previously described (Oyama, 2015). Briefly, sterile pin replicators (Molecular Devices Ltd., Berkshire UK) were used to transfer 2 μl metagenomic clones onto LB agar plates that had been plated before with 500 μl (OD_600 nm_ = 1) of pathogens such as *S. aureus, E. coli, Sal. Typhimurium, E. faecalis* and *L. monocytogenes*. Plates were incubated at appropriate temperatures for 24 h and zones of clearing around the clones were used to identify clones with inserts encoding antimicrobials. Putative antimicrobial positive fosmid clones were sequenced using Roche's 454 pyrosequencing platform. BLASTN (v2.2.28) on NCBI and BioEdit (version 7.1.11) (Hall, 1999) were used to edit and trim the vector sequence from the contigs. VecScreen on NCBI was used to search the sequences for vector contamination. Open reading frames (ORFs) were determined using the NCBI ORF finder program (Wheeler et al., 2003) and all ORFs with homology to antimicrobial genes and/or peptides were collated (Table 1). Based on expression levels and final protein yield, an ORF composed of 71 amino acids and named buwchitin was further investigated. Here, we report the activity of buwchitin.

**Table 1:** ORFs with homology to antimicrobial (biosynthetic) protein coding genes in rumen metagenome fosmids. All ORFs are from contig 1 of each fosmid and are in the 5′-3′ direction.

**Fosmid plate ID/ORF**	**Gene name**	**Protein size (AA)**	**Most similar homolog (*e*-value)**	**Putative function**	**Identity (overlapped AA)/% similarity**
SABPL5 C17/11	Gene 6	184	*Prevotella ruminicola* 23 WP_013063463.1 (3e-104)	4′-phosphopantetheinyl transferase family protein Synthesis of unusual molecules including polyketides, atypical fatty acids, and antibiotics	140/184(76%)
			*Butyrivibrio crossotus* CAG:259 WP_021960962.1 (2e-33)	Putative biosurfactants production protein	58/161(36%)
SABPL12(1) C3/9	Gene 17A	350	*Prevotella* sp. CDD20257.1(0.0)	3-dehydroquinate synthase DHQS represents a potential target for the development of novel and selective antimicrobial agents	250/346(72%)
SABPL12(1) C3/50	Gene 17B	80	*Pseudomonas putida* S16 NP_744149.1 (1.4)	Colicin V production protein	19/61(31%)
SABPL27 L10/66	Buwchitin	71	*Streptomyces mobaraensis* WP_004942604.1 *e*-value 5.0	Penicillin amidase Penicillin biosynthesis and metabolism	16/43(37%)
SABPL27 L10/73	Gene 68	68	*Ornithinibacillus scapharcae* YP_004810705.1 *e*-value 8.4	beta-lactam antibiotic acylase Penicillin biosynthesis and metabolism	22/63(35%)

### Amplification of antimicrobial genes

Extracted fosmid DNA (1 μl) from a metagenomic clone containing the buwchitin insert was used as template for PCR amplification. The buwchitin sequence was deposited in the GenBank database with accession number KY823515 and predicted to contain a signal peptide, when analyzed on the SignalP 4.1 server (Petersen et al., 2011). Primers were designed to start and stop at the first predicted methionine and at the last stop codon, respectively in order to conserve the reading frame and take account of the entire gene of interest. The primers used for the amplification of buwchitin gene were 5′-ATGAGGCTGTCACACGTTTG-3′ (forward primer) and 5′-TCACCAATCTGTATGGCACCG-3′ (reverse primer). Primers were diluted to a stock concentration of 100 μM and a total volume of 50 μl PCR reaction was set up as follows: 2 μl DNA template, 1 μl each of the forward and reverse primers (2 μM final concentration), 39.5 μl molecular grade water and 1 μl Titanium® *Taq* DNA Polymerase (Clonetech- Takara Bio Europe/SAS, France). *Taq* polymerase was activated for 1 min at 95°C, followed by 30 cycles of 95°C for 30 s, 68°C for 1.5 min, followed by a final extension step at 68°C for 1.5 min. PCR products were verified by electrophoresis on a 1.5% agarose gel using a 1 kb DNA ladder. Gel image was taken after exposure to UV using the Gel Doc™ XR^+^ system (BIO-RAD Hertfordshire, UK). Subsequently, the band of interest was excised with a sterile scalpel under a Dark Reader blue transilluminator (Clare Chemical Research Inc. USA) and DNA was purified and eluted using the QIAquick Gel Extraction Kit (Qiagen, Crawley, UK) according to manufacturer guidelines.

### Cloning of buwchitin gene and confirmation of cloning reaction

Cloning of buwchitin was carried out using the pTrcHis TOPO® TA Expression kit as described by the manufacturer. Five positive colonies from the transformation were analyzed for correct size, sequence and orientation of the insert. Selected colonies were cultured overnight in LB medium containing 100 μg/ml ampicillin and 0.5% glucose, and analyzed by PCR. Briefly, aliquots (1 ml) were lysed by heating for 10 min at 95°C in sterile 1.5 ml microcentrifuge tubes. The cell debris was pelleted by centrifugation at 13,000 × *g* for 2 min. The supernatant was used as template for the subsequent PCR. The PCR was set up in a total volume of 50 μl as follows: 2 μl of template DNA, 1 μl of gene specific forward primer (5′-ATGAGGCTGTCACACGTTTG-3′) and vector specific reverse primer (5′-GATTTAATCTGTATCAGG-3′), 21 μl molecular grade water and 25 μl MyTaq™ Red Mix (Bioline, UK Ltd., London UK). Initial Taq activation was performed at 95°C for 1 min, followed by 35 cycles of 95°C for 15 s, at insert specific annealing temperature for 15 s with an extension step at 72°C for 10 s, and a final extension step at 72°C for 7 min and holding at 4°C. PCR products were verified by electrophoresis on a 1.5% agarose gel using a 500 bp DNA ladder. A positive PCR control was also prepared using the control PCR template (expected size of 750 bp) and primers provided with the pTrcHis-TOPO® expression kit. Positive transformants were further analyzed by Sanger sequencing using plasmid DNA from extracted positive transformants as templates. The Xpress™ Forward sequencing primer for pTrcHis-TOPO® (5′-TATGGCTAGCATGACTGGT-3′) was then used to sequence the insert and alignments to original sequence orientation was confirmed using BioEdit (Hall, 1999).

### Expression and purification of his-tagged buwchitin

A single recombinant *E. coli* colony from a clone confirmed as containing the buwchitin gene was inoculated into LB broth containing 100 μg/ml ampicillin and grown overnight at 37°C with aeration and agitation (225–250 rpm). The following day, 1 L of LB broth containing 100 μg/ml ampicillin was inoculated with 20 ml of the overnight culture and incubated at 37°C under aeration (225–250 rpm). Gene expression was induced at OD_600 nm_ = 0.6 with 1 mM IPTG. Cells were harvested after 4 h by centrifugation (3,000 × *g* for 10 min at 4°C) and cell pellets were stored at −80°C for subsequent protein purification. Simultaneous purification and concentration of the buwchitin protein was carried out under native conditions using the Amicon® Pro Purification System (Merck Millipore Ltd Carrigtwohill, Ireland) following the manufacturer's protocol. Protein concentration was calculated as the ratio of absorbance at 280 nm [BioTek's Epoch™ Multi-Volume Spectrophotometer, (BioTek Instruments, Inc. Vermont, USA)] to the extinction coefficient absorbance (Abs 0.1% = 1 g/l calculated using the ExPASy ProtParam tool) (Gasteiger et al., 2005).

### Determination of minimum inhibitory concentration (MIC) of buwchitin

Vancomycin, Polymyxin B sulfate and ciprofloxacin were purchased from Sigma-Aldrich (Poole, Dorset, United Kingdom). All stock solutions were dissolved in the appropriate solvent prior to dilution in sterile distilled water (Andrews, 2001). MICs of buwchitin was measured by broth microdilution method using two-fold serial dilutions of antimicrobial agents in MH broth (CLSI, 2012). Buwchitin or comparator agents, vancomycin hydrochloride, polymyxin B sulfate and ciprofloxacin were added to the wells of a 96-well plate containing bacteria from overnight culture (adjusted to 1 × 10^8^ CFU/ml) to achieve a final inoculum concentration of 5 × 10^5^ CFU/ml (Cherkasov et al., 2008; Wiegand et al., 2008). MIC was defined as the lowest concentration of test agent that inhibited visible growth of the organism after 18–24 h of incubation at 37°C.

### Bactericidal/bacteriostatic activity of buwchitin

The bactericidal or bacteriostatic activity of buwchitin against *E. faecalis* was measured at MIC concentration using optical density measurements. An increase in both cell mass and cell number can readily be estimated by measuring the turbidity of a cell suspension using a spectrophotometer, thereby offering a rapid and sensitive alternative to cell counting (Dalgaard and Koutsoumanis, 2001; Madrid and Felice, 2005). This method has been shown to produce comparable results to plate counting, flow cytometric and green fluorescence viability analyses methods (Lehtinen et al., 2006). In a 96 well plate, buwchitin was added to cells in mid-logarithmic phase (1 × 10^6^ CFU/ml, OD_600 nm_ of ≤ 0.2) in MH broth and serially diluted as previously described. Plates were incubated at 37°C in a microplate incubator shaker. Wells without antimicrobial agents were used as growth control while wells with MH broth alone served as negative control. The rate of kill was calculated as a percentage (OD_600 nm_) of surviving cells over a 24 h period (Lehtinen et al., 2006; Hazan et al., 2012). The percentage of viable cells was normalized to 100% for the growth control (cells without antibiotic treatment).

### Erythrocyte leakage assay

The ability of buwchitin to lyse red blood cells was assessed in a 96 well plate using defibrinated sheep blood (Oxoid Ltd Hampshire, UK). Sheep red blood cells (RBC) washed and diluted (4%) in phosphate buffered saline (35 mM PBS) (pH 7.3) were treated with buwchitin at different concentrations and incubated at 37°C for 1 h. Triton X-100 (0.1% causes 100% cell lysis) served as a positive control. Absorbance (OD_450 nm_) of the supernatant (70 μl) from each well of the plate was measured to detect hemoglobin leakage from the erythrocyte cytoplasm and obtained results were used to determine the percentage hemolysis given that the 0.1% Triton X-100 represented 100% lysis after normalizing auto-hemolysis (PBS only treatment).

### Inner membrane depolarization assay (diSC3(5) method)

The ability of buwchitin to disrupt the electrochemical potential across the bacterial cytoplasmic membrane was measured by determining the amount of the membrane-associated probe, 3,3′-dipropylthiadicarbocyanine iodide [diSC_3_(5)] released from the cytoplasm (Wu et al., 1999; Lee et al., 2004). Briefly, mid-logarithmic phase (OD_600 nm_ = 0.2) *E. faecalis* cells were washed and resuspended to an OD_600 nm_ of 0.05 in 5 mm HEPES-glucose buffer, pH 7.2. In a 96-well plate, the cell suspension was incubated with 100 mM potassium chloride (KCl) and 0.4 mM 3,3′-dipropylthiadicarbocyanine iodide [diSC_3_(5)] until a stable reduction of fluorescence (excitation λ 622 nm, emission λ 670 nm) was achieved (~1 h). The KCl was added to equilibrate the cytoplasmic and external K^+^. After 1 h, buwchitin, positive control agent (0.1% Triton X-100) or negative control agent (untreated cells) were added to the cells in the wells. The plate was further incubated at 37°C with shaking while fluorescence was continuously monitored (excitation λ 622 nm, emission λ 670 nm) upon addition of peptide at 2–5 min intervals for 2 h.

### Transmission electron microscopy (TEM)

Exponential phase cultures of *E. faecalis* grown in MH broth were washed and resuspended to an OD_600 nm_ of 0.2 in 10 mM PBS. The cell suspensions (1 ml) were incubated at 37°C with buwchitin at 1 × MIC concentration in microcentrifuge tubes. To investigate possible changes in cell morphology following exposure to buwchitin, samples were removed at 1 and 24 h after exposure and prepared for TEM as previously described (Huws et al., 2013). Briefly, samples were fixed with 2.5% (v/v) glutaraldehyde, after which they were post-fixed with 1% (w/v) osmium tetroxide. Fixed samples were then stained with 2% (w/v) uranyl acetate and Reynold's lead citrate and observed using a JEOL JEM1010 transmission electron microscope (JEOL Ltd, Tokyo, Japan) at 80 kV.

### Molecular modeling of peptide 3D structures

Structural modeling of buwchitin was completed using the PHYRE2 web portal (Kelley et al., 2015). Results were visualized using the PyMOL v1.7.6 program (Schrödinger, 2010). The biophysical properties of buwchitin were predicted on the antimicrobial peptide database (APD2) (Wang et al., 2009).

### Statistical analysis

Two-way analysis of variance (ANOVA) with factors “antimicrobial treatments” and “time” was performed to determine whether there were significant changes in cell viability and membrane depolarization before and after treatment (Harmon, 2011). This was followed by *post-hoc* multiple comparisons using Tukey's HSD (Honestly Significant Difference) test (Bender and Lange, 2001; Feise, 2002; Harmon, 2011). Alpha (α) levels were set at *P* < 0.05.

## Results

### Sequencing, cloning, expression and purification using *In vivo* expression systems

**Figure 1:**
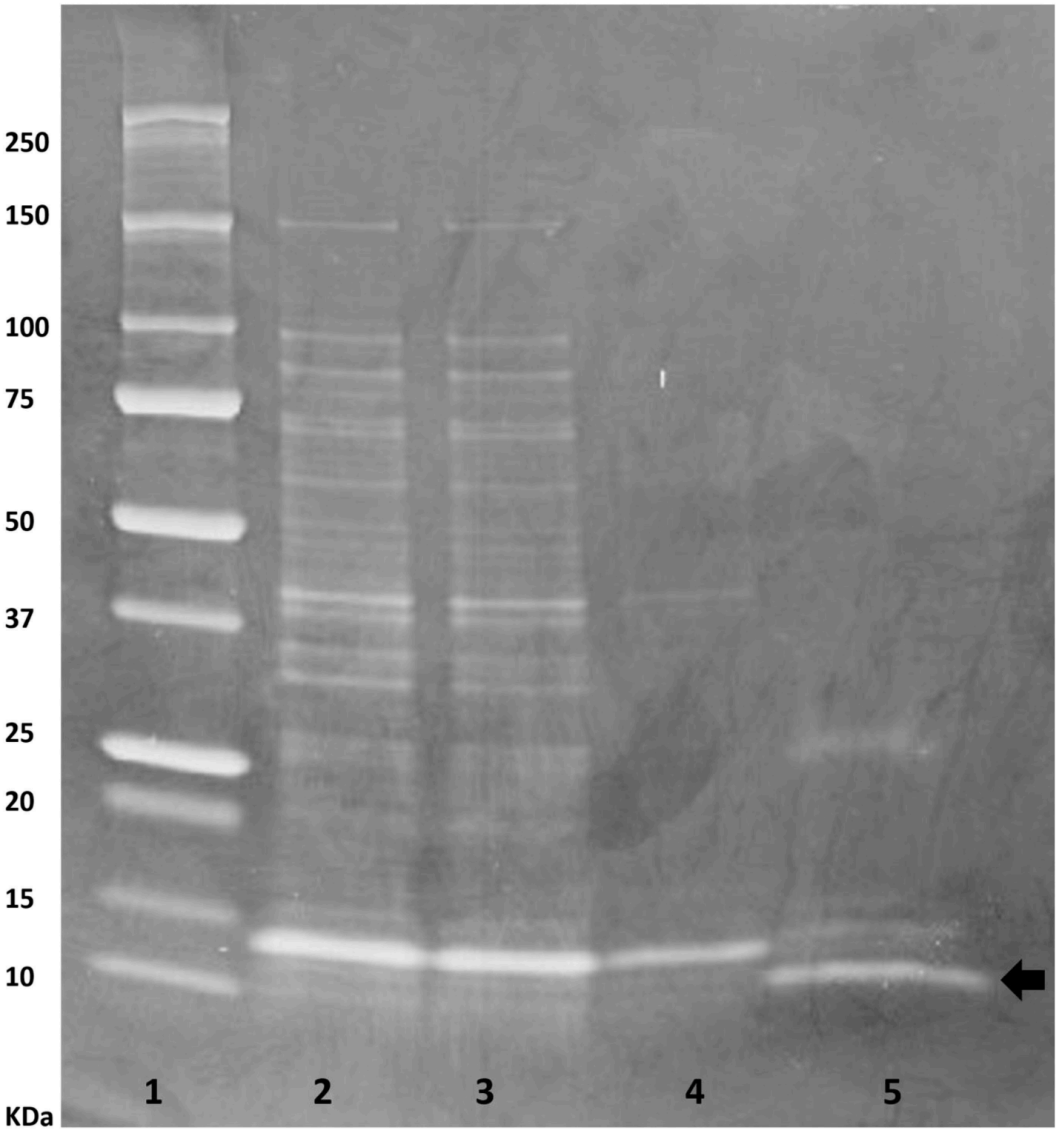
SDS-PAGE analysis of purification steps of buwchitin protein expressed in *E. coli* TOP10 cells on a 20% denaturing polyacrylamide gel (4 h after induction with 1 mM IPTG). Lane 1, protein molecular weight marker; Lane 2, cell lysate; Lane 3, supernatant; Lane 4, Wash step; Lane 5, eluted buwchitin protein. The arrow indicates band of purified protein of interest. Expected size is 8.35 (±3–4 kDa from His-tag).

The buwchitin gene was successfully PCR amplified using DNA from the fosmid clone, SAB PL27 L10/66. Bands of the correct size (expected size of 216 bp) were excised from the gel before proceeding to cloning. Electrophoresis results confirmed that the transformants carried the gene of the correct size, which was also confirmed by Sanger sequencing. The antimicrobial protein was expressed with an N-terminal 6xHis-Tag in *E. coli* to facilitate purification and investigation of its biochemical properties. Preliminary protein expression assay indicated that protein expression was optimal 4 h after induction (data not shown). SDS PAGE analysis of negative expression control (*E. coli* Top10 cells without plasmid) showed no protein expression bands while positive expression control (*E. coli* Top10 cells with pTrcHis-TOPO/lacZ) showed expression of the protein with a correct size of 40 kDa (data not shown). Cultivation of buwchitin transformants were scaled up to a total volume of 1 L to produce cell pellets for protein purification. Recombinant proteins were purified in their native conditions to preserve their activity (Karakus et al., 2016). Figure 1 shows the SDS-PAGE analysis of the purification fractions for buwchitin. The purification protocol reproducibly yielded a total of ~0.8 mg of purified protein per liter of culture.

### Antimicrobial and cytotoxic activity of buwchitin

**Figure 2:**
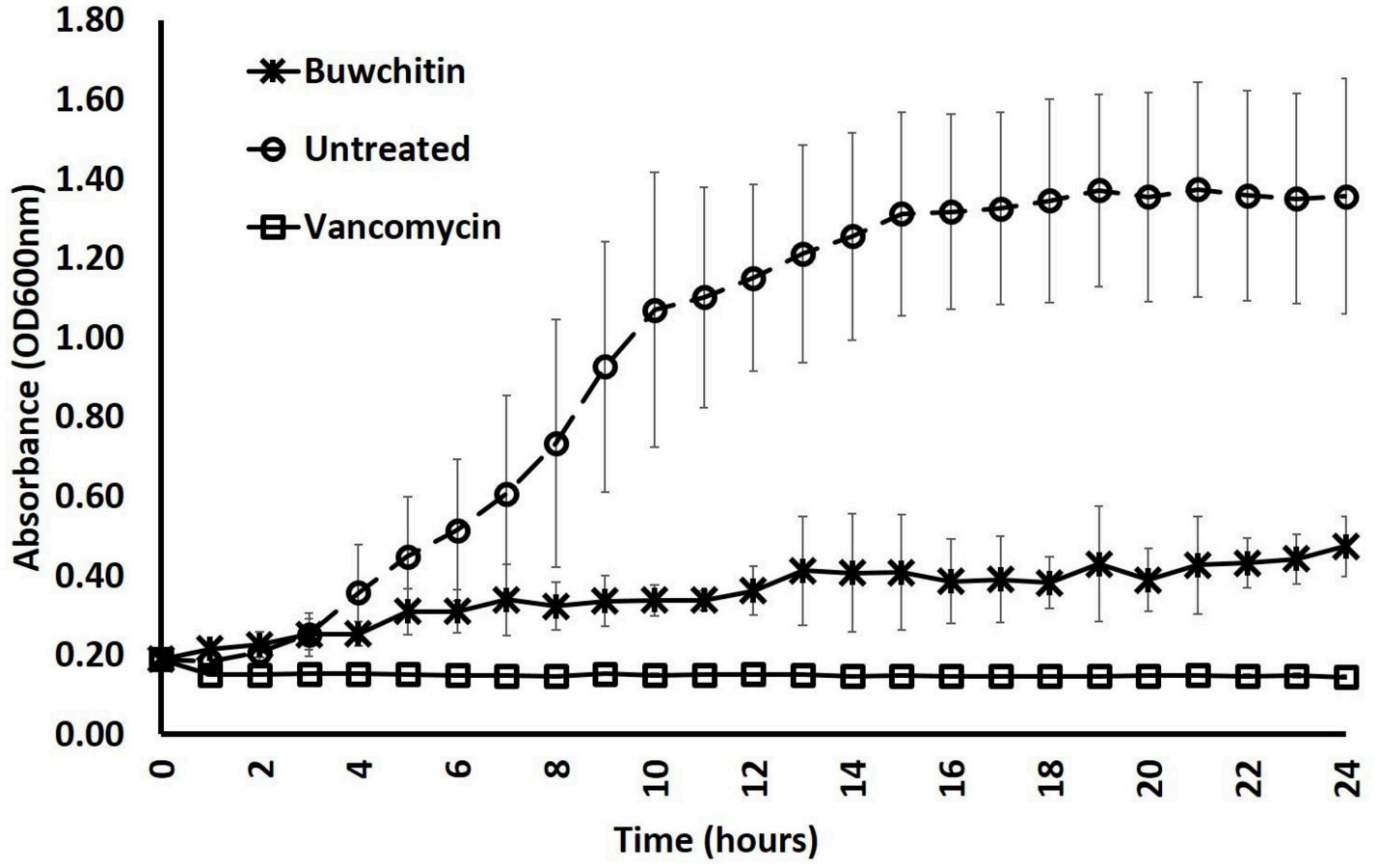
Growth rate of *E. faecalis* in presence of antibacterial agents. Growth rate was determined by monitoring cell density at OD_600 nm_ in three independent measurements at 1 × MIC concentration. Error bars represent the standard deviation.

Buwchitin was active against *E. faecalis* with an MIC of 100–200 μg/ml (Table 2). It also showed some inhibition of *E. coli* growth (observed in growth curves), but no detectable MIC at the highest concentration tested. This may account for the low level of expression of buwchitin in the *E. coli* expression host. The highest concentration of buwchitin tested was 400 μg/ml due to low levels of protein expression and/or yield of purified protein. The killing activity of buwchitin against *E. faecalis* was calculated as a percentage (OD_600 nm_) of surviving cells compared to the growth control. Only about 30 ± 1.4% surviving *E. faecalis* cells remained after a 24 h incubation period (*P* < 0.05). It would seem that buwchitin had a bacteriostatic effect against *E. faecalis* cells (Figure 2) as no change in *E. faecalis* cell density was observed over an incubation period of 24 h. Very little hemolytic effect (12.81 ± 0.02%) was observed when sheep red blood cells were treated with buwchitin at a concentration twice as high as the MIC determined for *E. faecalis* (Table 3).

**Table 2:** Minimum inhibitory concentration (MIC) of buwchitin and comparator antimicrobial agents (*n* = 6), > (precedes the highest concentration tested).

**Peptide ID**	**MICs (μg/ml)**			
	***Sal. typhimurium***	***E. coli***	***S. aureus***	***E. faecalis***
Polymyxin B sulfate	1.95	1.95	250	31.25
Ciprofloxacin	0.12	0.06	>250	62.5
Vancomycin hydrochloride	250	125	0.98	62.5
Buwchitin	>400	>400	>400	100–200

*Highest concentration of buwchitin tested is 400 μg/ml due to low protein yield*.

**Table 3:** Hemolytic activity of buwchitin against sheep erythrocytes. Sheep erythrocytes resuspended and diluted (4%) in PBS were treated with buwchitin (at different concentrations) or 0.1% (v/v) Triton X-100 and hemolysis was monitored at OD_450 nm_ at 1 h after incubation at 37°C, (values from three independent replicates and showing the standard deviation).

**Concentration (μg/ml)**	**% hemolysis**
400	12.81 ± 0.02
200	9.69 ± 0.09
100	5.23 ± 0.08
50	4.12 ± 0.06
25	4.15 ± 0.06
12.5	3.08 ± 0.03
6.25	2.80 ± 0.02
3.125	3.11 ± 0.06

**Figure 3:**
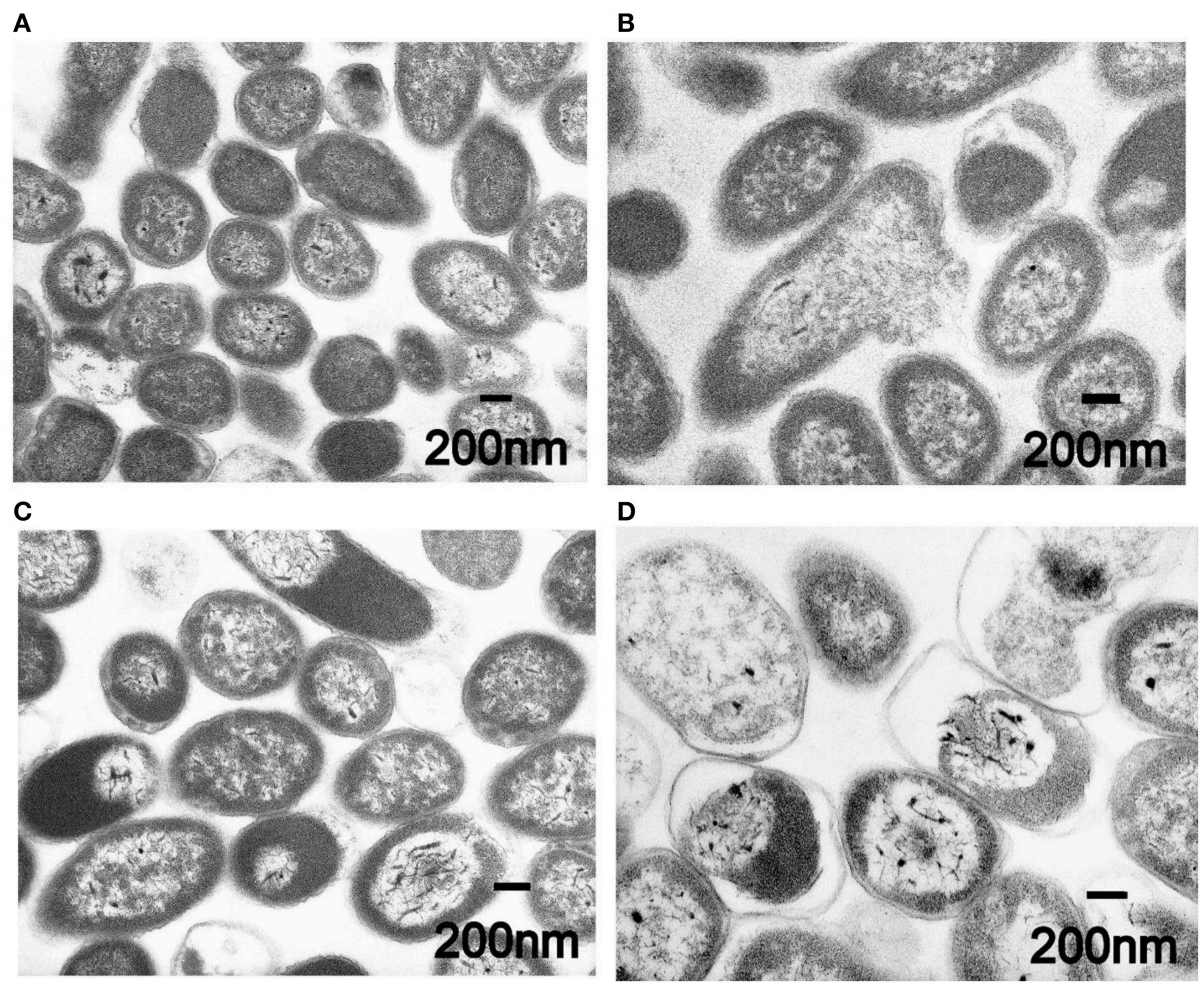
Representative transmission electron micrographs of *E. faecalis*. **(A)** Untreated *E. faecalis* cells at 1 h. **(B)** Buwchitin treated cells (200 μg/ml) at 1 h. **(C)** Untreated *E. faecalis* at 24 h. **(D)** Buwchitin treated cells (200 μg/ml) at 24 h. Scale bars on micrographs.

Buwchitin did not induce membrane depolarization in *E. faecalis* in the first 2 h of treatment. To determine whether the loss of viability in *E. faecalis* following exposure to buwchitin was accompanied by or was a result of changes in cell morphology and cell wall ultrastructure, TEM was performed. Electron micrographs of untreated *E. faecalis* at 1 and 24 h reveal intact healthy cells. Electron micrographs of buwchitin treated *E. faecalis* cells at 1 h showed intact outer membranes with blebbing but no major damaging effects and cell morphology changes. In contrast, micrographs of buwchitin treated *E. faecalis* cells at 24 h revealed several changes in cell morphology including cell lysis and detachment of the cell interior from the cell envelope (Figure 3).

### Structural modeling of buwchitin

**Figure 4:**
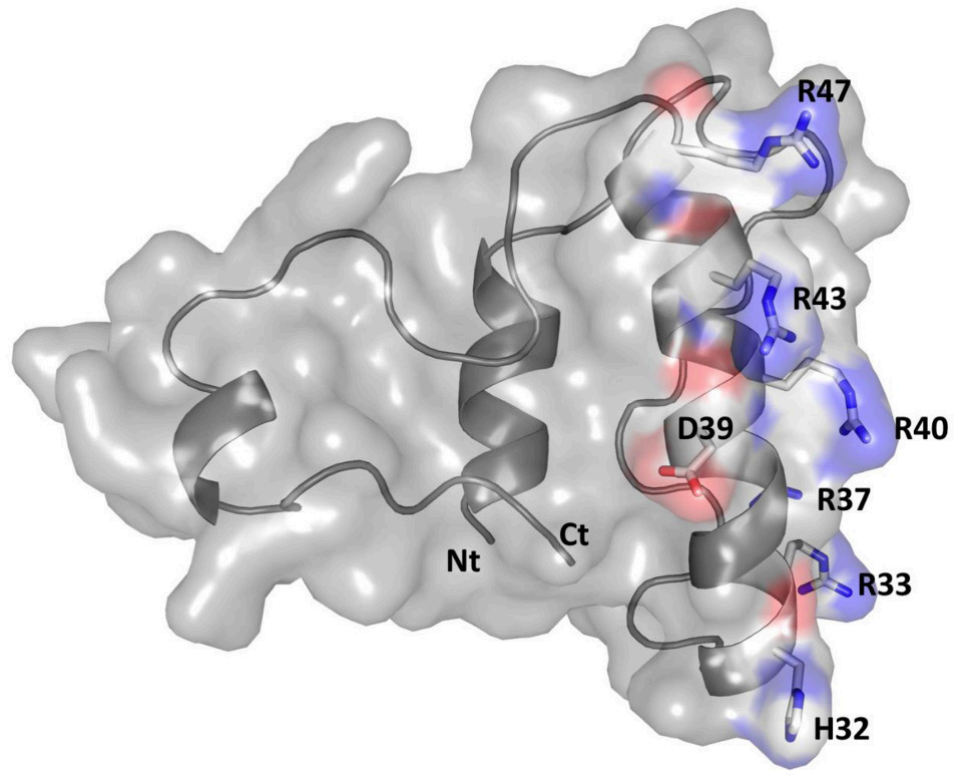
Structural model of buwchitin (gray) in cartoon and surface representation. Side chains of selected amino-acid colored according to atom type (N: blue; C: white; O: red). The N- terminus (Nt) and C- terminus (Ct) is also shown. Figure prepared using PyMol (Schrödinger, 2010).

Modeling and visualization of the 3D conformation of buwchitin using PHYRE2 (Kelley et al., 2015) and PyMOL v1.7.6 (Schrödinger, 2010), respectively, suggested that buwchitin is composed of a compact, all-helical, structure with major amphipathic helix connecting two smaller helices (Figure 4). The amphipathic helix agrees with a common structural feature of AMPs as the dual hydrophilic/hydrophobic nature allows the interaction and embedding of cellular membranes (Hancock and Sahl, 2006). As predicted by the APD2 database, buwchitin (71AA) is positively charged (+9), has a total hydrophobicity ratio of 29% and total Arginine and Lysine ratio of 19%.

## Discussion

Many currently used antibiotics were discovered by screening soil microorganisms that can be grown in the laboratory using standard microbial techniques for their antimicrobial activity (Ling et al., 2015). However, as natural product resources are practically inexhaustible, and approximately 99% of all species in external environments require more complex growth conditions than those provided using standard cultivation techniques, the majority of the world's microbial biodiversity remains to be explored (Harvey, 2007; Berdy, 2012; Lewis, 2013). Several recent studies already suggest that new organisms such as uncultured bacteria are likely to harbor new antimicrobials (Degen et al., 2014; Doroghazi et al., 2014; Gavrish et al., 2014; Wilson et al., 2014) and underexplored complex microbial communities, including the rumen, very likely represent rich sources of novel antimicrobials. These microbiomes have the potential to revive the platform of natural product discovery in a new culture-independent perspective, unbiased by the culturing aptitude of microbial species (Lewis, 2012; McCann et al., 2014; Kang et al., 2015). The potential for application of metagenomics to biotechnology seems endless as functional screens can be used to identify new enzymes, antibiotics and other biological agents in libraries from diverse environments (Gillespie et al., 2002; Lorenz and Schleper, 2002; Piel, 2002; Voget et al., 2003; Berdy, 2012).

In this study, we used a combination of functional and sequence based metagenomic screening strategies to prospect the rumen microbiome for novel antimicrobials as both strategies present advantages and limitations (Uchiyama and Miyazaki, 2009). Whereas, sequence homology based analysis allows for the identification of new enzymes from a range of environments, it requires a certain sequence similarity to members from known gene families, therefore limiting novelty. Functional screening of metagenomic libraries on the other hand, does not depend on previous sequence knowledge and therefore has the potential to discover novel classes of genes coding for desired functions without depending on their sequence similarity to already known genes (Ferrer et al., 2009; Simon and Daniel, 2009). We identified a novel antimicrobial gene, buwchitin, from the rumen microbiome and sought to express and characterize its antimicrobial activity against *E. faecalis*. Firstly, a fosmid-based cow rumen metagenomic clone library created from the solid attached bacteria of rumen content was functionally screened for antimicrobial activity. Clones with antimicrobial activity were subsequently sequenced to identify genes potentially involved in the antimicrobial activity observed in functional screens. Buwchitin, which was identified as a potential antimicrobial gene, was then expressed and further tested for antimicrobial activity. Buwchitin is a cationic (charge of +9), α-helical peptide (as predicted by 3D modeling), 71 amino acids in length and has a molecular weight of 8.35 kDa. Expression of buwchitin yielded on average 0.8 mg of purified protein per liter of culture. This relatively low yield may be due to the inhibitory effects of buwchitin on *E. coli* growth. However, this yield falls in the range reported in literature where concentrations of 0.5–2.5 mg/ml (Guerreiro et al., 2008), and 0.8 mg/ml (Zorko et al., 2009; Pei et al., 2014) were retrieved from 1 L cultures by different approaches using Ni-NTA columns. It may be useful to explore alternative expression systems, such as *Pichia* sp. or *Aspergillus* sp. to improve the yield of the protein.

Buwchitin was active against *E. faecalis* JH2-2 with an MIC of 100–200 μg/ml. This MIC is high when compared to antimicrobial proteins isolated and expressed using similar methods in other studies (Zorko et al., 2009; Elhag et al., 2017). Buwchitin (at MIC concentration) inhibited growth of *E. faecalis* cells with no change in *E. faecalis* cell density over a 24 h incubation period and has a minimum bactericidal concentration (MBC) of 200–400 μg/ml, suggestive of a bacteriostatic killing activity. Although most antimicrobial peptides are bactericidal (Hancock, 2001; Reddy et al., 2004; Lohner, 2017), many examples of bacteriostatic antimicrobial peptides exist in literature (Mine et al., 2004; Choi et al., 2016). For example, the human β-defensin 2 (hBD-2) is bacteriostatic against *S. aureus* only at concentrations as high as 100 μg/ml (Harder et al., 1997; Jung et al., 2011). Another example of a bacteriostatic antimicrobial peptide is the human lactoferricin (LfcinH) (Gifford et al., 2005). Furthermore, most antibacterials are potentially both bactericidal and bacteriostatic depending on bacterial pathogen (Pankey and Sabath, 2004). Further investigations into the mechanism underlying the bacteriostatic action of buwchitin would be necessary to come to a final conclusion about its accurate classification. Buwchitin had minimal hemolytic activity against sheep erythrocytes, suggesting that buwchitin may have selective activity against microbial cells. Despite these encouraging results, it will be necessary to carry out cytotoxicity assays on human and other mammalian cell lines to determine whether buwchitin can induce apoptosis and necrosis in cells (Paredes-Gamero et al., 2012). Very little or no membrane depolarization was observed in *E. faecalis* cells treated with buwchitin and TEM images of buwchitin treated cells showed intact outer membrane and very little changes in cell morphology after 1 h of treatment. Only after 24 h of treatment were large vacuoles in the cytoplasm and separation of the cell envelop observed. Given the low depolarizing activity of buwchitin, it would seem that membrane-destabilizing activity alone does not explain the antimicrobial activity of buwchitin. It is known that poly-cationic AMPs bound to teichoic acids including lipoteichoic (LTA) and wall teichoic acids (WTA) build a poly-anionic ladder and may initiate bacterial killing by facilitating the entry of peptides into the cytoplasmic membrane without membrane depolarization (Schneewind and Missiakas, 2014; Malanovic and Lohner, 2016). Further investigation into buwchitin teichoic acid binding and other mode of action studies are required to gain insights into its mechanism of action and the events leading to cell death.

Buwchitin is positively charged and has an amphiphilic structure with 29% hydrophobic residues as has been observed for many antimicrobial peptides (Hancock and Sahl, 2006). This positive charge greatly facilitates the accumulation of AMPs at the polyanionic microbial cell surfaces and may be sufficient for antimicrobial action (Hancock and Sahl, 2006), thus perturbing the membrane integrity. Some cationic peptides have been shown to translocate or form multimeric transmembrane channels promoting the membrane depolarization, which seems to contribute to their activity (Shai, 1999; Bhattacharjya and Ramamoorthy, 2009) at higher concentrations. The amphipathic nature of the predicted peptide structure and the observations in the TEM images is in agreement with this type of interaction, indicating that although buwchitin is not membrane destructive, it may interact with components of the cell envelop such as the enterococcal polysaccharide antigen. The formation of vacuoles in the cytoplasm also appear to support this idea. Still, at the current stage, it remains difficult to say which of the known membrane interaction and disruption models (i.e., barrel stave, carpet models, or micellar aggregate model) explains the activity of this peptide without further experimental evidence.

Further studies remain to be performed to enhance the antimicrobial phenotype of buwchitin. One potential strategy to improve the antimicrobial activity of buwchitin is the pepscan technology, in which shorter active fragments and optimized amino acid substitutions and/or modifications are identified by a scanning approach. These active peptide fragments identified by pepscan can then be SPOT-synthesized on cellulose membranes and systematically screened for antimicrobial activity (Hilpert et al., 2007; Winkler et al., 2009). The use of pepscan mapping and SPOT arrays has been shown to be useful for simultaneous optimization of peptides to generate new sequences that possess a variety of therapeutic and biological properties (Chico et al., 2010; Haney et al., 2015; Merino-Gracia et al., 2016; Ortega-Villaizan et al., 2016). Peptide improvements that might result from the pepscan technology might provide buwchitin derivatives with greater antimicrobial activity, similar to what has been achieved for other peptides in the literature (Knappe et al., 2016; Mikut et al., 2016). An evaluation of MICs against a panel of different bacterial species and *in vitro* stability studies in the presence of plasma or serum would also be beneficial. To explore the possible therapeutic relevance of buwchitin, further *in vitro* cytotoxicity studies and *in vivo* studies with acute toxicity in mice at concentrations above the MIC would be required.

In conclusion, the data we generated and present here suggest that we discovered a novel rumen protein, buwchitin, with potential antimicrobial properties. It is furthermore possible that with substantial modification, this AMP might qualify as a potential antimicrobial agent for the treatment of *E. faecalis* infections, which would favor further investigation of the protein. This study also highlights the enormous value of prospecting the rumen microbiome, and other microbial communities for novel compounds to expand our limited antimicrobial drug toolbox.

## Author contributions

LO and SH conceived the project. LO and JC completed the laboratory work under supervision of SH, JE, and CC. SG and LO completed the sequencing and downstream analysis of the sequences, respectively. AC and NF helped LO with transmission electron microscopy and 3D structural modeling, respectively. FP, OG, and PG created the rumen fosmid metagenome library. MH, KH, CC, and HM have provided valuable ideas into the project from conception. LO wrote the paper with input from all co-authors.

### Conflict of interest statement

The authors declare that the research was conducted in the absence of any commercial or financial relationships that could be construed as a potential conflict of interest.
